# The development of play face mimicry: comparative insights from human infants, adults and orangutans

**DOI:** 10.1098/rstb.2025.0132

**Published:** 2026-09-24

**Authors:** Tonko Willem Zijlstra, Christopher Riddell, Milica Nikolic, Mariska E. Kret

**Affiliations:** ^1^ Leiden University, Institute of Psychology, Cognitive Psychology Unit, The Netherlands; ^2^ Leiden Institute for Brain and Cognition (LIBC), Leiden, The Netherlands; ^3^ University of Amsterdam, Research Institute of Child Development and Education, The Netherlands

**Keywords:** mimicry, laughter, play face, emotional expressions, primates, affect

## Abstract

Laughter and its associated facial expression, the play face, are critically important in the social lives of animals. Perhaps owing to this, play faces are mimicked in social interactions across many species. Despite growing interest, few works have directly compared play face mimicry across species and age groups within the same experimental framework, limiting our understanding of its developmental and evolutionary origins. In this study, we examined play face mimicry in human infants (*n* = 63), human adults (*n* = 36) and orangutans (*Pongo pygmaeus; n* = 6), using a tightly controlled design. We found that both human infants and adults reliably mimicked play faces, however, infants produced these robustly more than adults. Additionally, while adult humans did not produce as many play faces as infants, they produced reliably more smiles in response to the play face stimuli. When considering the orangutans, we found no evidence of play face mimicry, with only one instance observed across the entire experiment. This suggests that while our paradigm reliably induced mimicry across both human groups, our set-up may have been suboptimal to evoke mimicry responses in orangutans.

This article is part of the theme issue ‘Mechanisms, development, phylogeny and functions of emotional expressions’.

## 1. Introduction

Laughter is a staple expression of joy and pleasure in many different species of animals. For humans, often not a day goes by that we do not laugh [1], and laughter plays an important role in a variety of daily social activities [2]. Additionally, in non-human primates, laughter is also contagious and plays an important role in social bonding [3–5]. As comparative and ontological studies are lacking, little is known about the similarities and differences between the mimicry of laughter displays in humans and other great apes, as well as between infants and adults. This current study aims to address this gap, focusing on the facial expression associated with laughter, and directly compares its mimicry in human infants, human adults, and six orangutans (*Pongo pygmaeus*): one juvenile and five adults.

### (a) Laughter and the play face across species

We define laughter as a multi-modal response that typically involves a vocalization characterized by short voiced and unvoiced notes (resulting in the distinctive ‘ha-ha’ sound), along with a facial expression involving relaxation of the facial muscles and the dropping of the jaw [6–8]. In non-human primates, laughter tends to be unvoiced and noisy [9]. The facial expression that accompanies laughter vocalizations is commonly referred to as a ‘laughter face’ in humans and a ‘play face’ in non-human animals [10–13]. Notably, previous research has revealed striking similarities between human infants and young children, and many non-human animals, both in the form of laughter expressions and the contexts in which they occur [10,14–16]. Henceforth, we therefore use the term play face consistently to describe this facial expression in both species, as this consistent terminology avoids misleading distinctions between homologous behaviours observed in humans and other animals [17]. Finally, in humans, the play face differs from the smile in both form and muscular involvement. Smiling typically involves only the upward pulling of the lip corners (and sometimes the raising of the cheeks), without the relaxation of the facial muscles or the dropping of the jaw that characterizes play face expressions [18].

In the literature, there exists some discussion about how the play face evolved and, importantly, how it relates to laughter and smiles in humans and the bared-teeth display in great apes (which we define as an expression in which the lips are retracted and the teeth are exposed, without a drop of the jaw) [19,20]. The play face probably evolved as a signal of non-threat during rough-and-tumble interactions; with an open mouth, relaxed facial muscles and rhythmic breathing helping to distinguish playful intent from aggression and to regulate arousal during social engagement [19,21]. It has traditionally been argued that human laughter faces are homologous to the primate play face and that human smiles have their origin in bared-teeth displays [19,22]. However, more recently, it has been suggested that smiles signalling positive affect may also have evolved from relaxed open mouth displays [20]. While the exact evolutionary origins of human laughter and smiles are debated, it seems evident that there are many similarities between smiles and laughter in both function [8,23–26] and form [27–29].

In non-human primates and human infants, play faces are mostly bound to rough-and-tumble play contexts, such as chasing, play fight and tickling episodes ([10,30], but see [20,31]). These expressions are often spontaneous and closely tied to the dynamics of physical interaction, capturing the immediate emotional and social processes that unfold during play [32]. By contrast, human adult play faces occur in more varied ritualized contexts, often decoupled from strict physical play. Adult laughter emerges in a broad variety of contexts and social situations—when we hear a funny joke, out of politeness or in negative contexts, such as when we observer the others’ misfortune (i.e. schadenfreude) [6,33,34]. Laughter and play face production also often happen after hearing someone else laugh or seeing them produce a play face, a phenomenon known as mimicry [3,10,34–39], although the study of play face mimicry in humans specifically is lacking. We define mimicry as an automatic and matched behavioural response that follows from the perception of a similar action or expression by another individual [10,35,36,40].

Using primarily naturalistic experiment-like methods, previous studies have examined play face mimicry across all other great ape species—bonobos [41], chimpanzees [42,43], gorillas [4] and orangutans [44]—as well as several other species (e.g. geladas [45], meercats [46] and sun bears [47]). These studies investigated whether play faces, observed in naturally occurring play bouts, were more likely to occur after the playing partner had produced a play face compared to scenarios in which no play face had yet been produced, while controlling for play intensity as well as facing direction [42,44]. Results from these works suggest that great apes (and many other species) produce and mimic play faces during playful interactions with conspecifics. Subsequent studies have highlighted that this phenomenon is enhanced by face-to face contact between playmates [4,41]. However, playback studies of laughter vocalizations, which commonly co-occur with play face displays [44,48,49], have shown that chimpanzees do not mimic these vocalizations [50,51]. The fact that mimicry has been observed in studies focusing on play bouts, whereas vocalizations are not reliably mimicked in playback studies, suggests that social context may play an important role in whether a behaviour is mimicked [52,53]. It remains unclear whether mimicry occurs more readily when individuals are actively engaged in playful interaction compared to situations in which live social interaction is absent. Investigating play face mimicry in situations where individuals are passive observers, rather than active participants, may help clarify the functional boundaries and prevalence of mimicry in non-human animals.

Importantly, mimicry of the play face may help prevent playful interactions from escalating into bouts of aggression [10,54]. In this case, mimicry may act as a ‘social glue’ [55–57] facilitating smoother social interactions in a variety of species—including meerkats [46], geladas [45], macaques [58], humans [59,60], chimpanzees and lowland gorillas [4]. Notably, research has shown that in both chimpanzees and gorillas, it is specifically the mimicry of play faces—not merely the presence of the play face—that is associated with longer play bouts [4]. This may be explained by the fact that mimicry makes one more similar to one's interaction partner, and this has been argued to allow individuals to better predict the behaviour of others through the embodiment of their facial expressions [40,61–64]. In addition to this important intra-personal role, mimicry may also serve as a communicative tool, confirming that the signaller's playful intent has been accurately perceived, thereby reinforcing mutual engagement and signalling a shared willingness to continue the interaction [3,10,58].

### (b) The development of (play face) mimicry

Despite a myriad of works on mimicry in human adults (for reviews, see [36,37,63,65]), there has been comparatively little research done on the development of mimicry [66–68]. Regarding the studies of mimicry in human ontogeny, the precise age at which human infants begin to mimic facial expressions is controversial. While early works suggested that neonates in the earliest days of life could mimic several expressions [69,70], more recent investigations have challenged this position [71–73]. Possibly, a lack of appropriate control stimuli left open the possibility that ‘mimicked’ behaviours are a result of processes other than mimicry (e.g. arousal or affect) [74]. As such, the most recent evidence seems to suggest that facial mimicry can be reliability demonstrated later in infancy than seminal works originally suggested. After the neonatal period, mimicry of positively valanced facial expressions appears to be systematically demonstrable from approximately seven months old [75–78], broadly coinciding with the period that infants begin to discriminate between distinct emotions [79]. Additional work indicates that infants mimic the facial expressions of others at an even younger age, from five months, when congruent visual and auditory stimuli are presented [80]. Regarding the mimicry of laughter vocalizations in infancy, only a few studies exist. In one study [67], the authors examined mother-infant laughter over the first two years of life while mothers freely interacted with their infants. The authors examined the extent to which laughter vocalizations were produced reciprocally (i.e. with no overlap between partners, which would indicate some form of mimicry) or concomitantly (i.e. with overlap). Surprisingly, laughter in mother-infant dyads was found to follow neither of these patterns but rather occurred most frequently in isolation, suggesting that laughter in early infancy may not necessarily be mimicked but could reflect infants’ independent emotional expressions or independent moments of positive affect.

Regarding the development of mimicry in non-human animals, there is evidence in a number of primate species that both infants and juveniles exhibit facial mimicry [41,45], and this response appears to develop across ontogeny [3,43,44]. A seminal study [44] found that four out of five participating infant orangutans mimicked the open-mouth faces of their interaction partners during live dyadic play bouts. In another study of infant chimpanzees, aged 12 to 15 months, the authors found that the mimicry of play faces during social interactions occurred above chance and at a rate that increased significantly with age [43]. An additional study on play faces and vocalizations in chimpanzees found that, compared to juveniles and older individuals, only one infant in the sample mimicked a conspecific's play expression during play, also suggesting that, although present in early development, such mimicry becomes more frequent across age [3]. Contrastingly, in another study on mimicry in juvenile rhesus macaques in a semi-naturalistic setting, mimicry rates were found to decrease across juvenile development [81], although these authors examined a range of both affiliative and non-affiliative behaviours. In other primate species, such as bonobos [41] and geladas [45], mimicry of facial expressions appeared to emerge already during infancy (although see [74]). Together, these findings suggest that mimicry may be adaptive and confer important intra- and interpersonal benefits from early in life [81]. This, in turn, underscores the importance of a comparative approach that examines mimicry across age groups and species to better elucidate its developmental and evolutionary roots.

### (c) The present study

The current study investigates the mimicry of play faces and associated smiling behaviours in response to observed play face expressions. Mimicry plays a central role in social cognition and there has long been interest in understanding its ontogenetic and phylogenetic origins across species [4,6,82–84]. Although theoretical frameworks suggest that play face mimicry serves important intra-personal and social functions in both humans and non-human primates, empirical comparative studies remain limited. To address gaps in our understanding of how mimicry develops over the lifespan, particularly regarding play face expressions, this study includes both human infants and adults performing a comparable highly controlled mimicry task, allowing us to assess the extent to which such expressions are shaped by learning and socialization. Importantly, the individuals in the stimuli did not make sustained eye contact with the participant, allowing us to limit the extent to which the participant may have felt as if they were involved in the interaction. Therefore, we were able to examine whether mimicry would occur in the absence of a live social interaction, extending previous work that has demonstrated mimicry in such contexts.

Orangutans make a good candidate species for investigating the phylogenetic origins of mimicry as they are the great ape species most distantly related to humans [20] and are, amongst all great ape species, the least social [85]. Evidence of mimicry in all three groups would suggest that this is a trait that not only develops early in ontogeny but is also one that arose early in our evolutionary history. If evidence of play face mimicry previously observed in naturalistic settings can be replicated in a setting without live interaction in this predominantly solitary species, this would strengthen the claim that although mimicry can have social consequences, it also has more direct benefits for the mimicking individual [40,83]. Differences between humans and non-human animals, in turn, may help pinpoint when mimicry probably arose in our phylogenetic history, providing insight into its evolutionary importance. By comparing human infants and adults, we can examine whether the capacity for mimicry changes across ontogeny. This can shed light on how, and to what extent, social capacities for emotional mimicry develop and are influenced by socialization and learning. Together, this approach bridges developmental and comparative perspectives, allowing us to examine the continuity of play-related facial expressions from our great-ape relatives to early human development and into adulthood. Our design targets the intersection of ontogeny and phylogeny by asking not only whether mimicry is present across species but whether its expression changes across development in a manner similar for both species. If mimicry is present very early across groups but varies in, for example, timing, frequency or context sensitivity across age classes; this could be consistent with developmental shaping of a potentially conserved mechanism. If age-related patterns differ across species, this could be consistent with an important role for social ecology and learning in modulating the mimicry response.

To this end, 15-month-old human infants, human adults, one juvenile and five adult orangutans engaged in a passive viewing task in which we measured their propensity to mimic the play faces of the individuals in the stimuli. Participants viewed video clips of dyads of conspecifics engaged in social interaction in two conditions: (i) displaying play faces (experimental condition); or (ii) neutral expressions (control condition). Based on previous work in human adults [57,86], human infants [77,80] and orangutans [44], we expected to find more occurrences of play faces in experimental trials compared to control trials, which would suggest mimicry of these expressions. Since smiling may be seen as a precursor to laughter and occurred commonly in humans in response to the stimuli we presented, we also examined participants’ smiling behaviour in response to the play faces presented in the videos, and whether this differed across groups. To ensure direct comparability, we also examined the bared-teeth displays produced by orangutans, as these have been argued to be homologous to human smiles [19].

## 2. Methods

This methods section provides a summary of the most essential parts of the methodology. Please see the supplementary material S1 for a complete and detailed description.

### (a) Participants

Participants were *n* = 36 human adults, *n* = 77 15-month-old human infants and *n* = 6 orangutans. See table 1 for an overview of the demographic information for human and orangutan participants and table 2 for a detailed overview of orangutan participants. Adult participants were mostly students and were recruited in pairs via the Leiden University research participation website (SONA) and rewarded with research credits or €3.50 for their participation. Infants were recruited and tested at the University of Amsterdam, as part of a longitudinal study on the development of the social self and rewarded with a toy and a ‘Young Scientists’ certificate for their participation.

**Table 1.** Demographic information.

group	*M*_age_	s.d*.*_age_	age range	*%* female
human adults	26.09 years	12.48	19–66 years	69.4%
human infants^a^	14.91 months	0.69	12–16.31 months	40.42%
orangutans^b^	39.33 years	13.70	5–45 years	50%

^a^ Only *n* = 47 parents filled in the demographic questionnaire.

^b^ See the supplementary material table S2 for more detailed information on the orangutan individuals.

**Table 2.** Demographic information of orangutan participants. (Note: this table provides an overview only of the orangutans that participated in the study. The actual subject pool was much larger, as orangutans were not coerced or forced in any way to participate.)

subject	sex	age (years)	zoo	*n*_trials_
Amos	male	21	Apenheul	5
Anak	female	45	Ouwehands	10
Kevin	male	40	Apenheul	18
Sabar	male	5	Ouwehands	17
Sandy	female	39	Apenheul	10
Wattana	female	26	Apenheul	32

Orangutans were housed at two different zoos in The Netherlands: Apenheul (*n* = 4) and Ouwehands Dierenpark (*n* = 2). Although there was a total of 15 orangutans housed at both locations, only six of these individuals were interested in participation. Orangutans at both sites were habituated to visitors and familiar with researchers. Before we conducted our experiment, a training period was conducted to familiarize the orangutans with the research set-up. Participation was strictly voluntary: individuals were never forced, rewarded or separated from their groups.

#### (i) Data exclusions

A total of *n* = 14 infants were excluded from the experiment owing to fussiness or technical errors, therefore the final sample size was *n* = 63 infants. As indicated in detail below, *n* = 4 adult participants correctly identified the aims of the study, and we decided to run the analyses with these participants included and excluded, which did not ostensibly change our results. Given this, we report the results of our analyses with these participants included.

### (b) Design

Since we analysed human and orangutan data separately, the design of the study was slightly different across groups. In humans, the present study had a between-subjects design with one between subjects factor: group membership (two levels: human adult and human infant) and one within-subjects’ factor: stimulus type (two levels: control stimulus (in which no play or laughter face was visible) and experimental stimulus (in which a play or laughter face was clearly visible)). In the orangutans, since we did not compare responses between juveniles and adults, we retained only the one within-subjects factor, namely stimulus type. Owing to the differing attentional capacities of each group, participants completed a different number of trials. Human adults completed *n* = 32 trials, human infants completed *n* = 8 trials and orangutans completed an average of *n* = 15.33 trials (see the supplementary material, S1; table S3 for trial breakdown).

### (c) Materials

#### (i) Human adults and infants

The stimuli used in the present study were four different 4-second video fragments gathered from the Dutch public television network (NPO) and YouTube. Stimuli consisted of two children engaged in an interaction with one another, presented in full colour without sound. In experimental trials, two children were shown smiling and at least one child displayed a smile with a jaw drop (i.e. a play face: action units (AUs) 12 + 25 + 26). In control trials, the children showed only neutral facial expressions.

#### (ii) Orangutans

The stimuli used for the orangutan portion of the study were four different 4-second video fragments gathered from YouTube. Several primer videos (e.g. of wild animals, a man cleaning a window) were shown before the experimental trials to attract the orangutan's attention and motivate them to sit in front of the screen. Stimuli consisted of two to three juvenile individuals engaged in interaction with a conspecific, presented in full colour without sound. In experimental trials, at least one individual displayed a play face (AUs 10 + 12 + 25 + 26). In control trials, the individuals showed only neutral facial expressions, although in one control trial an individual also displayed a jaw drop (AUs 25 + 26).

### (d) Procedure

#### (i) Human adults and infants

The experiment with human adults took place at the laboratories of Leiden University. Participants were provided with an information brochure and were asked to provide written consent. Because participants may have modified their behaviour if they were privy to the aims of the experiment (measuring facial mimicry), in the information brochure they were simply told that the experiment aimed to measure their ‘attention’ while viewing video scenes.

**Figure 1.**
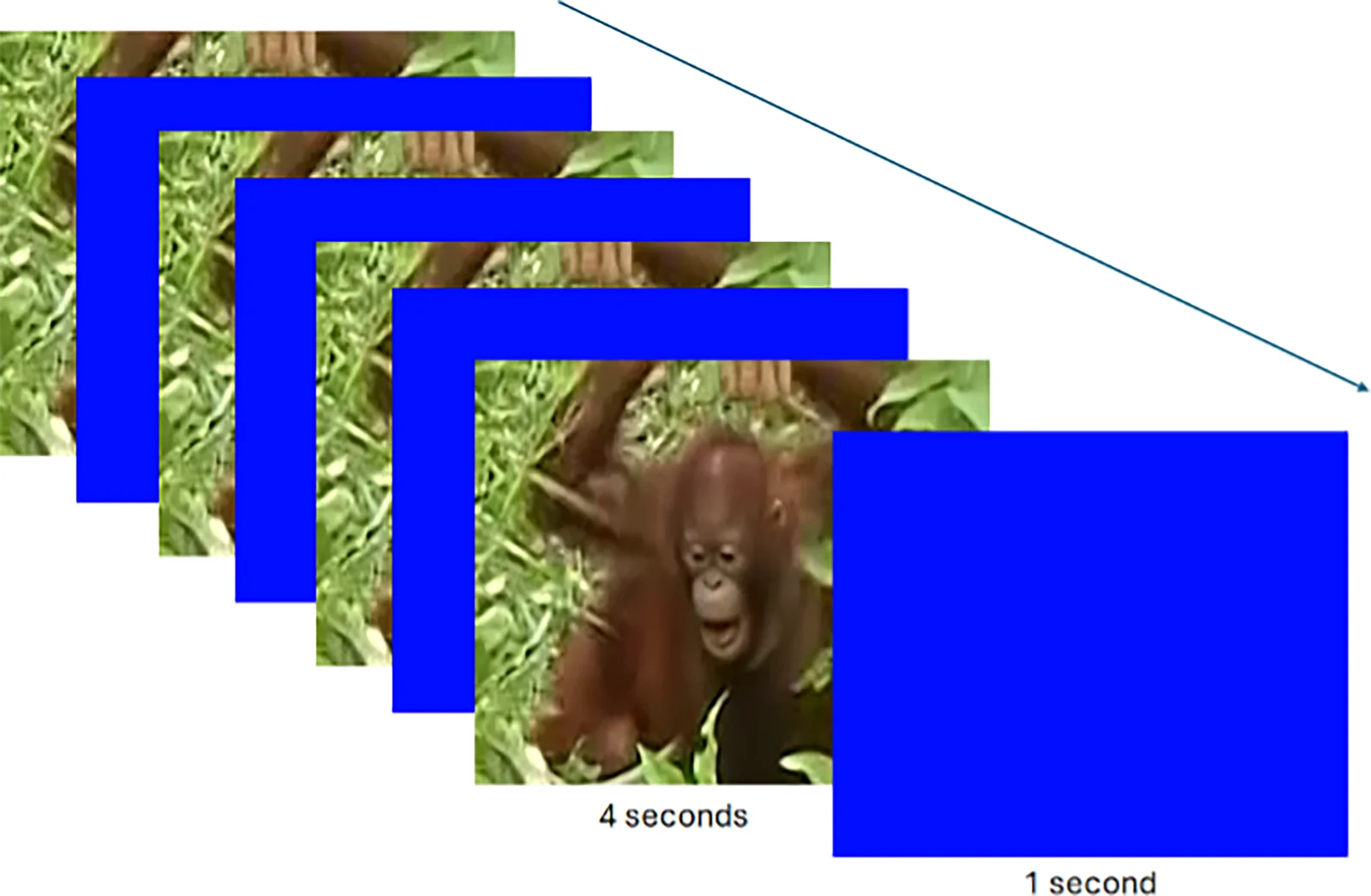
Trial structure of experiment. Note: each trial (in all three experiments) consisted of four repetitions of the same 4-second-long video fragment followed by a 1-second-long presentation of a blue screen, for a total trial length of 20 seconds.

Human adults saw a total of *n* = 32 trials, divided into four blocks of *n* = 8. The same stimuli were displayed in each block, but the order of control and experimental trials was counterbalanced across blocks. Each block contained an equal number of control (*n* = 4) and experimental (*n* = 4) trials. In each trial, one of the eight stimuli was displayed and repeated four times (figure 1). After viewing all the stimuli, the experiment was concluded. Since deception was used, the researcher asked each participant whether they were aware of the aims of the study and noted any participants that indicated this. For human infants, the procedure was very similar. The mother and infant pairs were asked to sit in front of a computer screen and view one block of trials identical to those used in human adults. After viewing the eight trials, the experiment was concluded. In total, this took approximately 4 minutes.

#### (ii) Orangutans

To begin the experiment, the television screen was placed in front of one of the enclosures. If the individuals in said enclosure were not interested or unwilling to participate, the screen was moved to the next enclosure. The screen was easily visible and accessible to all subjects. To ensure the focal individual's face was visible, the experiment was started only if no play or feeding behaviours occurred within the preceding five minutes.

After an orangutan approached the television screen, one of the primer videos was displayed. If the individual sat in front of the screen for more than three uninterrupted seconds, an experimental trial began. Unlike in the human experiment, a trial for orangutans consisted of one video display (control or experimental), followed by an observation period of 3.5 minutes. As with the human experiment, a single trial consisted of four iterations of the same short segment (4 seconds), with a blue screen interspliced between each segment (1 second). Therefore, each trial took approximately 19 seconds to complete. Individuals participated in a maximum of six trials per day, with a minimum break of 30 minutes after two consecutive trials. A video of an example trial is provided in the supplementary material, S3.

### (e) Statistical analyses

#### (i) Scoring behaviours

Human behaviours were coded manually trial-by-trial by a certified FACS coder (C.R.). The first authors T.W.Z. and C.R. coded 20% of the total videos together to ensure sufficient inter-rater reliability, which was good (Cohen's kappa > 0.77) for all behaviours. Orangutan behaviours were coded by author T.W.Z. with the help of a student research assistant. Behaviours were coded as present or absent in a particular trial. Of central relevance to our research question was the play face, in humans, which we defined as a smile (activation of the lip corner puller, *zygomaticus major*, AU12), combined with a parting of the lips (AU25) and dropping of the jaw (AU26). In line with previous work on orangutans, we defined a play face as activation of AUs 10 + 12 + 25 + 26 (or 27) and a bared-teeth display as activation of AUs 10 + 12 + 25 [87]. We only coded a play face as present when it occurred directly in response to the viewed stimulus material, excluding any instances that appeared spontaneous or potentially occurred owing to seeing the facial expression or behaviour of another individual in the testing environment (e.g. a human infant's mother or another orangutan conspecific).

#### (ii) Modelling

We performed all analyses using R Studio version 3 [88]. To analyse the presence of play faces and smiles (which were coded as 1 = ‘present’ and 0 = ‘absent’ in any given trial), we ran a series of Bayesian generalized linear mixed models with Bernoulli distributions in the *brms* package [89]. We ran all models with four chains and 5000 iterations (including 1000 warm-up iterations). As per the recommendations of Depaoli & van de Schoot [90], we examined model convergence by visual inspection of the trace plots and histograms of the posteriors as well as inspection of the Gelman and Rubin diagnostics. Since we had only limited studies to base our prior estimates on (many of which used drastically different methodologies), we used the default *brms* priors for all models, flat priors for all fixed effects and Student's *t* priors with 3 degrees of freedom for the intercept and other variance parameters.

#### (iii) Human infants and adults

For human infants and adults, we constructed separate models for play faces and smiles, all of which had the same structure. We ran two models which included the effect of *condition* (experimental trial versus control) as a predictor of play faces and smiles. Secondly, since the stimuli used in the infant and adult study were identical, we decided to directly compare infants and adult mimicry behaviour in a single analysis. Here, we used the first eight trials of the adult data (which were identical to those presented to infants) and ran a model which included *condition* (experimental versus control) and *age* (infant versus adult) as predictors of play faces and smiling. This allowed us to compare any mimicry of play faces and smiles across age groups to examine any between-group differences. Finally, after comparing adults and infants directly, we ran two models which included the effect of *condition*, as well as the effects of other potentially influential behaviours as covariates (to examine whether the effect of *condition* became weaker or disappeared). Details of these control behaviours are highlighted in the ethogram in the supplementary material S1, table S4 and explained further in the Results, §3d ‘Control models’.

#### (iv) Orangutans

For orangutans, we approached the analysis differently. To foreshadow our results, there was only one orangutan play face display and zero bared-teeth displays, across all the valid trials. This made statistical analysis of our data unsuitable, and rather, we provide a descriptive account of our findings.

### (f) Transparency and openness

We report how we determined our sample size, all data exclusions (if any), all manipulations and all measures in the study, and the study follows JARS [91]. All non-identifiable data, analysis code and research materials are available at: https://osf.io/k9x36.

## 3. Results

### (a) Descriptives

Count data of all laughter face, smile and bared-teeth display observations, split across groups and trial type (experiment versus control) can be found in the supplementary material S2, tables S5 and S6 respectively). Human infants produced play faces in *n* = 179 trials (37.1%), human adults in *n =* 55 trials (4.8%) and orangutans in *n* = 1 trial (1.1%). Human infants produced smiles in *n* = 100 trials (20.7%), whereas adults produced these in *n* = 285 trials (25.1%).

### (b) Human play face and smile mimicry

We first began our analyses by considering infants and adults separately. All full models are displayed in the supplementary material, S2. When considering play faces in human infants, condition was a robust predictor of play face displays, *b* = 0.99, 95% credible interval (CI) = [0.52, 1.48], odds ratio (OR) = 2.68, probability of direction (*pd*) = 1.00 (supplementary material, table S7). When considering play face mimicry in human adults, the effect of condition was similarly robust, *b* = 0.91, 95% CI = [0.17, 1.67], OR = 2.46, *pd* = 0.99 (supplementary material, table S8). These data suggest that human infants and adults did reliably mimic play faces when they were displayed, although the effect appeared slightly larger in infants.

Furthermore, as indicated, we decided to also examine whether smiling occurred significantly more in experimental as compared to control trials. When considering smiles alone in human infants, condition was not a robust predictor of smiling, *b* = 0.02, 95% CI = [–0.48, 0.51], OR = 1.02, *pd* = 0.53 (supplementary material, table S9). Conversely, in human adults, condition was a robust predictor of smile behaviours, *b* = 0.84, 95% CI = [0.54, 1.15], OR = 2.32, *pd* = 1.00 (supplementary material, table S10).

**Figure 2.**
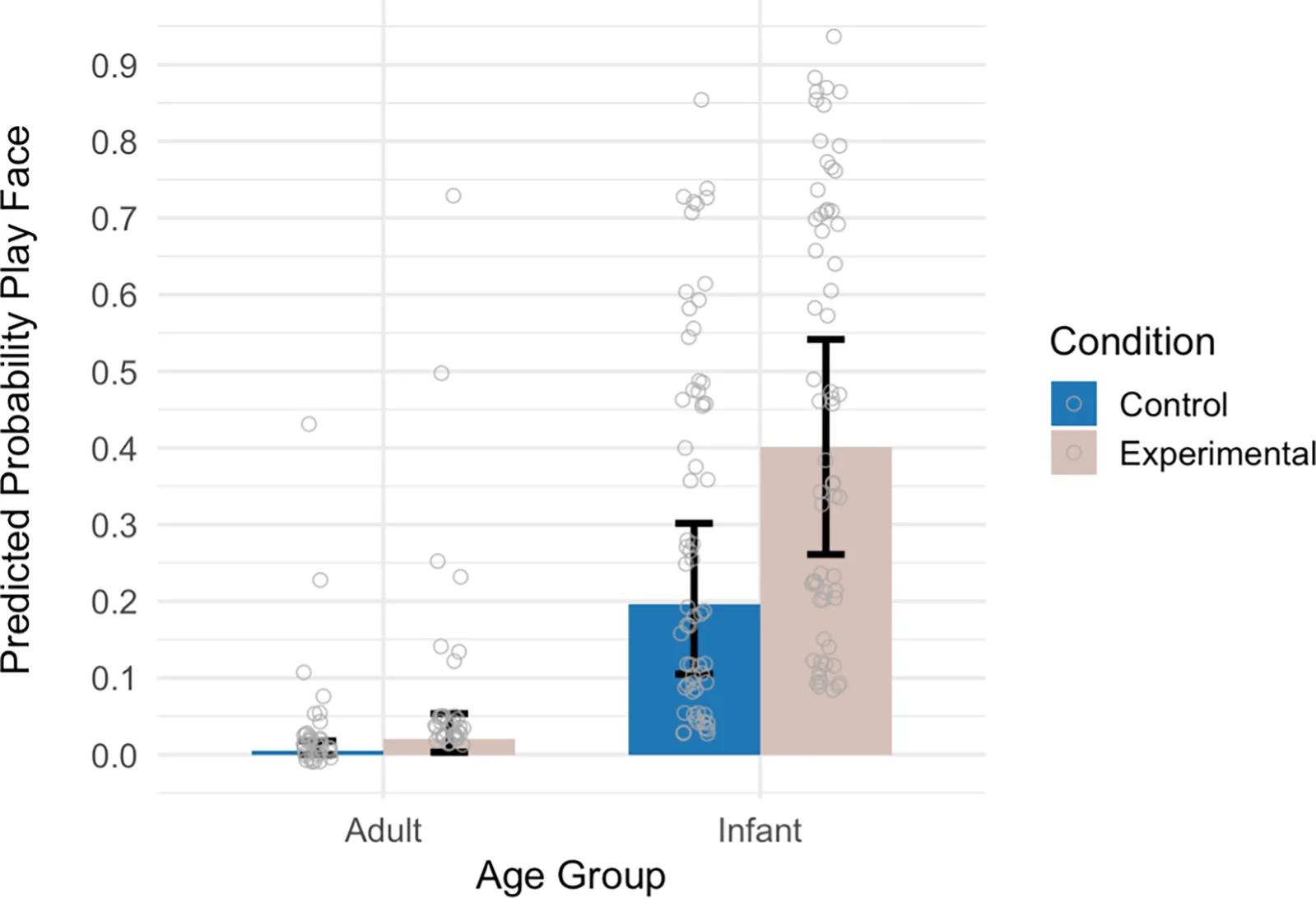
Probability of play face displays across human age groups and experimental conditions. Note: error bars represent 95% highest density posterior (HDP) intervals. Dots represent participant predicted probabilities averaged across *n* = 8 trials.

To follow up these individual models, we ran a model in which we compared infant and adult behaviour directly by using the first eight trials of the adult task (and all valid trials of the infant task). The model predicting play faces revealed that the condition and age group were meaningful predictors, *b* = 1.38, 95% CI = [0.10, 2.78], OR = 3.98, *pd* = 0.98; *b* = 3.84, 95% CI = [2.31, 5.62], OR = 46.73, *pd* = 1.00, respectively. The interaction between condition and age group was not a robust predictor of play faces (supplementary material, table S11). Examination of the variance decomposition revealed that approximately 17% of the variability in play face presence was owing to differences between participants. Ultimately, from this model, we could conclude that, across both groups, there was evidence of play face mimicry, given that the odds of the production of this display increased by a factor of 3.98 in experimental compared to control trials. Additionally, play faces were robustly more common in infancy no matter the condition, with the odds of an infant producing a playface increasing by a factor of 46.73 compared to adults, although there was clear variability between participants. This relationship is displayed in figure 2.

**Figure 3.**
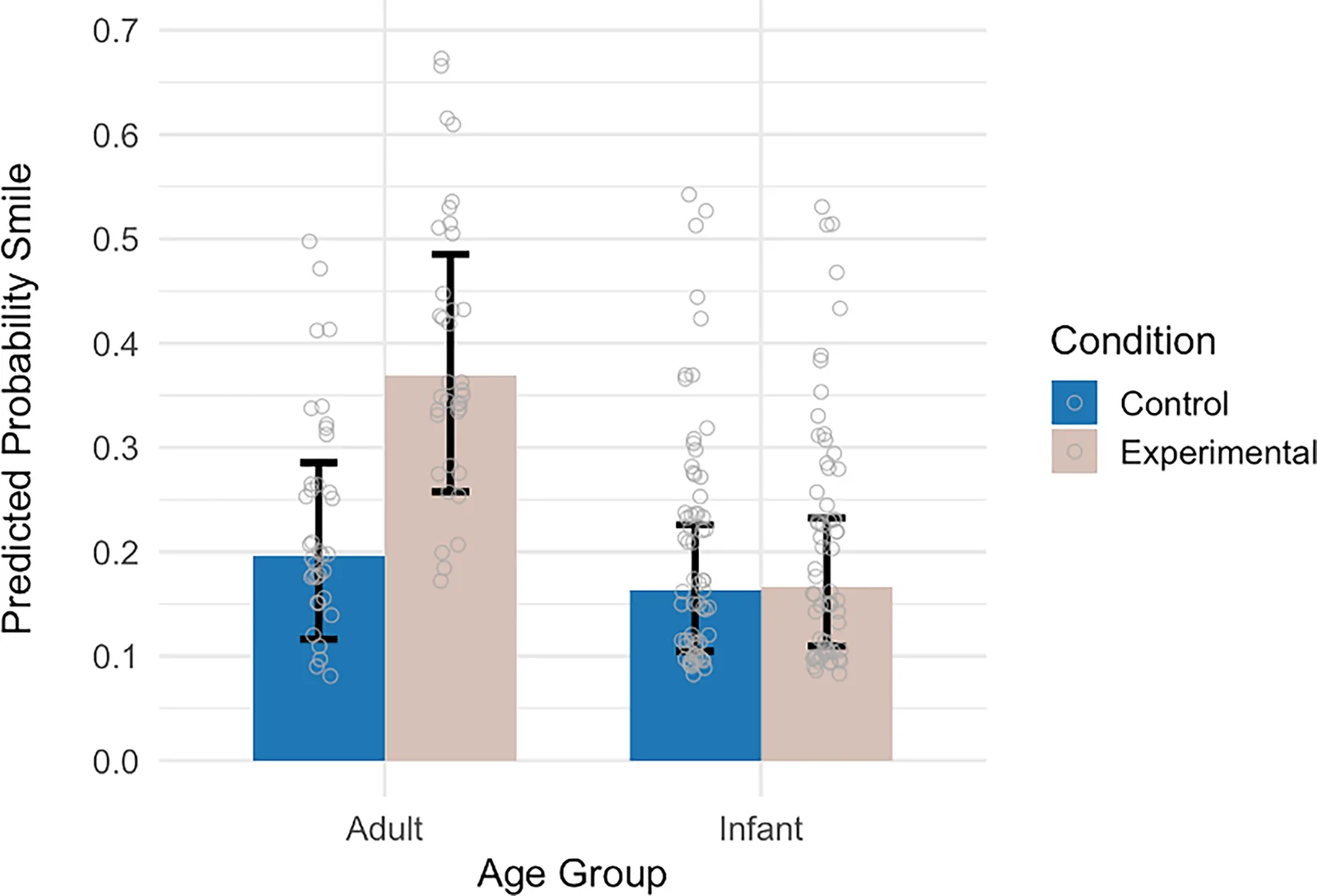
Probability of smile displays across human age groups and experimental conditions. Note: error bars represent 95% HDP intervals. Dots represent participant predicted probabilities averaged across *n* = 8 trials.

Conversely, the model on smile displays revealed a robust interaction effect between age group and condition, *b* = –0.86, 95% CI = [–1.60, –0.14], OR = 0.42, *pd* = 0.99 (supplementary material, table S12). As displayed in figure 3, while adults displayed robustly more smiles in experimental compared to control trials, infants displayed similar levels across trial types. The odds ratio suggests that the odds of infants smiling while viewing the experimental trials were 0.42 times the odds of adults smiling in the same condition. Examination of the variance decomposition revealed that approximately 10% of the variance in smiling behaviour was owing to between-participant differences. As such, while the experimental condition meaningfully increased the likelihood of adults’ producing a smile, this effect was much weaker for infants.

Ultimately, the results of both analyses suggest while both infants and adults mimicked play faces (albeit to differing degrees), only adults displayed more smiles in experimental compared to control trials.

### (c) Orangutan play face mimicry

In the orangutans, we observed only one single play face expression and no bared-teeth displays. The single play face was shown by Sabar and occurred in an experimental trial approximately 1 minute after the presentation of this video. Sabar was 5 years old at the time of testing, and the two playing individuals in the stimulus were also juveniles. However, it is important to note that the orangutans generally did not show great interest in the screen. An exact Wilcoxon rank sum test, comparing looking time in the play condition to looking time in the control condition, showed that there was no significant difference in the amount of time spent looking at the stimuli in control (*M* = 12.58) compared to experimental (*M* = 13.59) conditions, *Z* = –0.56, *p =* 0.578.

### (d) Control models

As indicated, there were several behaviours that could have influenced human participants’ propensity to mimic the stimuli displayed. For example, the presence of maternal positive vocalizations, maternal play faces and maternal pointing behaviours may have influenced human infants’ propensity to themselves produce play faces. Additionally, since human adults were tested in the presence of another participant, the likelihood of producing a play face may also have been influenced by characteristics of this other individual, such as their familiarity (known versus unknown), gender (male versus female) and whether that individual made eye contact with them.

To control for this, we ran models for human infants and adults separately which predicted play faces and smiles, while including these potentially influential behaviours as covariates. Full model outputs can be found in the supplementary material, S2. Examination of the human infant model revealed that the effect of condition on play face displays was robust and remained influential, but was slightly weaker when taking these additional covariates into account (*b* = 0.66, 95% CI = [0.15, 1.18], OR = 1.94, *pd* = 0.99; supplementary material, table S13). Additionally, in this model, mothers’ play face displays were also found to be a meaningful predictor of the infants’ play faces (*b* = 1.28, 95% CI = [0.68, 1.92], OR = 3.59, *pd* = 1.00). This indicates that the odds of infants’ producing a play face increased by a factor of 3.59 when mothers themselves displayed a play face. Importantly, this was not owing to infants’ mimicry of the parents’ facial behaviour, as we only considered play faces that occurred as a response to the viewing of the stimulus in this analysis (and not simply as a response to interacting with the mother or play faces that occurred spontaneously). No other covariates meaningfully influenced the occurrence of infants’ play faces (all 95% CIs spanned zero). Examination of the human adult model revealed that, when controlling for the additional explanatory variables, the effect of condition remained a meaningful predictor of the occurrence of adults’ play faces, *b* = 0.91, 95% CI = [0.17, 1.69], OR = 2.48, *pd* = 0.99 (supplementary material, table S14). No other variables robustly explained adults’ play face behaviours (all 95% CIs spanned zero).

As condition was not a robust predictor of smiling behaviour in infants, we performed the control model on smiling behaviours for human adults only. In this model, condition remained a meaningful predictor of smiling (*b* = 0.89, 95% CI = [0.57, 1.22], OR = 2.44, *pd* = 1.00; supplementary material, table S15). As with the play face control model, characteristics of the co-present individual seated next to them, such as familiarity, sex and whether that individual made eye contact did not influence smiling (all 95% CIs spanned zero). Ultimately, the results of these analyses suggest that our above findings hold when considering these potentially influential variables, indicating that these findings are robust.

## 4. Discussion

Play faces represent an important component of social life in many animals [7,10,15,19,92]. In both human and various non-human animals, play faces are integral to social play [30,93], and there is evidence that these displays are reliably mimicked in a number of social species [10,15]. In addition to its social communicative function, mimicry has been argued to serve the intra-personal function of helping the mimicker to better understand their interaction partner [56], potentially by fostering more efficient predictions about their behaviour [40,83]. In this case, mimicry may help one better understand the playful intentions of one's partner and adjust one's own behaviour accordingly. Unsurprisingly, thus, the interest in mimicry in the literature has been great [10,15,59,65,68,76]. However, previous works on mimicry, especially those using naturalistic interaction paradigms, also suggest that there may be other factors that influence the likelihood of mimicry occurring, like social context [41,42,81,83]. This has led to debates about the limits of mimicry, particularly in non-human animals and human infants [71,73,74,80].

In this experiment, we sought to examine play face mimicry in human adults, infants and orangutans. To do this, we used a controlled design in which participants viewed stimuli of interacting juveniles displaying play faces (experimental condition) and neutral faces (control condition). Since the individuals in the stimuli did not make sustained eye contact with the participant, we limited the extent to which the participant may have felt as if they were involved in the interaction. Additionally, despite a wealth of works on mimicry in adulthood in both the human and non-human animal literature, few works have considered whether there are developmental differences in mimicry by testing children and adults in the same task, which formed the second aim of our experiment. Importantly, this allowed us to examine whether there were any important developmental differences in the extent to which certain expressions of emotion were mimicked.

In line with previous works on emotional mimicry [36,57,77,80], we found that human participants reliably mimicked play faces, although there were clear individual differences in the extent to which participants displayed these facial expressions. Interestingly, although both infants and adults produced more play faces in experimental compared to control trials, indicating that mimicry had occurred, infants produced robustly more play faces overall. Contrastingly, we found no clear evidence of play face mimicry in our orangutan sample, with only one instance of a play face produced across all six individuals that participated.

When considering smiling behaviours in humans, adults produced more smiles in the experimental than in the control condition, suggesting that they responded to the positive emotion conveyed by the play faces, even without directly mimicking the exact expressions. In other words, some level of emotional contagion in response to our stimuli occurred [94]. Infants, by contrast, did not show this pattern. Additionally, orangutans did not produce bared-teeth displays, the assumed homologue of the human smile [19,22].

### (a) Ontogeny of mimicry and emotional contagion in humans

Both 15-month-old human infants and adults showed more play faces in experimental trials (in which a play face stimulus was visible) compared to control trials (in which only a neutral face was visible), suggesting that play faces were reliably mimicked in both groups. This result supports previous works on mimicry in human adults (for reviews, see [36,37,63,65]) and human infants [75–78]. In human infants, it has been found that facial expressions of emotion, such as smiles, are reliably mimicked from around seven months old [77] and at five months old when stimuli are presented using both auditory and visual cues [80]. Despite these claims, previous works with infants have predominantly focused on the mimicry of smiles without jaw drops [75,80,95] and laughter vocalizations [67,96]. In this study, we provide, to our knowledge, some of the first evidence that, in addition to these expressions, young infants also mimic the play faces of conspecifics. Additionally, as indicated, in our experiment, and in contrast to the works discussed above [77,80], the individuals depicted in our stimuli did not make sustained eye contact with the participants, and the interaction was not live. This distinction is important, as it suggests that, at least at 15 months old, eye contact and live interaction may not be necessary for the mimicry of facial emotions to occur, reflecting its potential intra-individual function in humans across ontogeny. However, further work will be necessary to test the boundaries of this condition. This result contrasts with a previous study on four-month-old infants which found that eye contact modulated the extent to which participants mimicked facial behaviours (e.g. mouth opening and eyebrow raise) [97]. De Klerk *et al.* [97] found that no mimicry occurred in the condition where the model showed averted gaze, which suggests that direct gaze was a necessary precursor for mimicry in human infants at this young age.

Interestingly, we found clear developmental differences in the extent to which play faces were expressed in human participants. While the presence of the mimicry of play faces was consistent across age in humans, infants produced robustly more play faces than adult participants. One explanation for this finding comes from work on social play, particularly regarding its frequency throughout the lifespan [32,94,98]. Importantly, the time devoted to play typically decreases throughout the juvenile years and into adulthood in both humans [99] and non-human animals [32]. It follows that play faces, therefore, may be produced more spontaneously and in higher frequency during infancy [100]. In adulthood, laughter (and its associated non-verbal expression, the play face) is much more ritualized and occurs in context-dependent ways, such as during conversations, out of nervousness or during humorous events [6,33]. The stimuli used in our experiment (playing juveniles) were perhaps more in keeping with the normal contexts in which laughter occurs in infancy, resulting in an overall higher rate of play face production in this group.

The finding of diminished play face responding in human adults contrasts the results we found when considering smiling behaviours. Compared to play faces, adults showed more smiles and especially so in response to the experimental videos. This finding is in line with a previous study on laughter vocalizations, in which it was found that many participants reliably smiled in response to audible ‘canned’ laughter (even though they may not necessarily have produced an audible laugh) [39,101]. Although one interpretation of this result is that smiling may have operated as a ‘low intensity’ version of laughter, we wish to add nuance to this. Importantly, previous work has suggested that while there are some overlaps [8,23–26], smiles and laughter may still serve different functions depending on the social context [6]. In particular, smiles may be more controlled, whereas laughter is often more spontaneous and linked to higher arousal [102]. Smiling behaviours in our group of human adults in response to the stimuli may indicate that some form of emotion contagion had taken place, although potentially at lower intensity [103,104].

To summarize, our results suggest that play faces are reliably mimicked in both human adults and infants. Additionally, while human adults displayed more smiles in experimental compared to control trials, this was not the case for human infants.

### (b) Phylogeny of mimicry in orangutans and humans

Interestingly, we found no evidence of play face mimicry in our orangutan sample, which consisted of six individuals, with only one play face produced across the entire experiment (produced in the experimental condition). Additionally, they did not produce any bared-teeth displays. Although this was a surprising finding, we wish to be careful about interpreting this as evidence for a lack of play face mimicry in orangutans. This result was somewhat unexpected, given the fact that several studies have shown that orangutans do tend to mimic facial expressions in some contexts [105], including play faces in live play contexts [44]. Although, it should be noted that a pilot study in which videos of play scenarios were shown to orangutans demonstrated that these stimuli similarly did not elicit play faces outside of the context of live play [20]. Interestingly, one study found that both infant and juvenile orangutans did mimic the play faces of conspecifics during dyadic play [44], providing a direct developmental and species-relevant comparison for the present work. In the case of our study, while most human infants did mimic play face displays, the single juvenile orangutan in our sample did not. Although this comparison is limited by the small sample size, this may provide further evidence of the critical role of live interaction for the elicitation of mimicry in orangutans.

While orangutans are clearly capable of mimicking some facial expressions, the fact that, unlike humans, they do not seem to do so under the specific conditions of this experiment, may be related to differences in the social structure of the two species. Although orangutans probably descend from an ancestor that, similarly to most primate species, lived in social groups, they now live semi-solitary lives [85]. It is therefore possible that, while they retain the fundamental traits necessary for (facial) mimicry, the tendency to do so, and therefore the frequency with which they do, has decreased. Alternatively, this tendency may have also become more exaggerated in humans (and potentially the *Pan* and *Gorilla* genera [3,4,106]). This notion is in line with findings which show that orangutans produce spontaneous laughter during play bouts much less often than the other non-human great apes [44,107]. These findings suggest that this signal may simply be less relevant to orangutans compared to other great apes.

Regardless of the relevance of the signal itself, it is also questionable whether it is useful for an individual to produce a facial expression when they are not involved in an interaction themselves [108,109]. However, it is easy to imagine that producing context appropriate facial expressions may still have some function beyond its potential benefits for the observer. Specifically, embodied expressions may lead to greater emotional understanding [62,110], while also playing a role in reputation management within the group setting. This may be the case even if the producer of the expression is not actually involved in any social interaction. In a very social species, such as humans and members of the genus *Pan*, understanding others’ mental states and third-party reputation formation is a common and important factor that impacts social relationships and hierarchy [111–114]. Contrastingly, the mimicry of facial expressions may be less useful, beyond the clear benefits for the expresser, if it is rare for there to be a third-party or audience present during any social interaction, as is the case in orangutans compared to other great apes [115,116].

It is, of course, also possible that orangutans sometimes mimic play faces, even when these are presented in video recordings. However, the conditions of our study may have made this unlikely. It may be that the presence of a positive, naturalistic setting is critical for eliciting play face mimicry in orangutans. For example, Leavens *et al.* [52] argue that, in addition to an over-reliance on WEIRD (Western, Educated, Industrialized, Rich and Democratic) samples in psychology to represent ‘humans’, research on zoo-reared orangutans can also be limited in ecological validity, as these individuals may have fewer opportunities for naturalistic social interactions. However, previous studies have demonstrated that zoo-reared orangutans can exhibit mimicry of behaviours, such as yawning and scratching, even when these are presented via video stimuli (e.g. [105,117]) and that they can also produce play face responses during live social interaction with conspecifics (e.g. [44]). Therefore, the absence of play face mimicry in our study is unlikely to be owing simply to the captive background of our participants or to the use of video stimuli *per se.* Instead, we propose that the contextual and emotional characteristics of our paradigm, particularly the lack of direct, reciprocal social engagement, may have constrained the expression of mimicry [53].

In addition to the setting of our study, the results obtained in humans revealed substantial individual variation in mimicry, as well as clear age-related differences in the frequency of play face behaviours. These findings suggest that the absence of mimicry in orangutans should be interpreted cautiously, as the smaller and older composition of the orangutan sample could have decreased the likelihood of observing mimicry at the group level. If we assume, hypothetically, that humans and orangutans do not differ in their general tendency to mimic play faces, having a higher proportion of juveniles in the orangutan sample could have increased the overall likelihood of observing mimicry (as has been found in previous works in both orangutans [44] and other great ape species [3,51,106]).

### (c) Limitations and future directions

Despite contributing to our understanding of play face mimicry and the production of emotional expressions in human and non-human animals, using a more tightly controlled design, there are some limitations worth mentioning. Firstly, as indicated, the number of orangutan participants (and completed trials) was low. It will be important for future studies to test a wider group of individuals to see whether evidence of play face mimicry can be found when more individuals are tested. Additionally, a further limitation is that we only tested infants at 15 months old and only one juvenile orangutan. As such, it was not possible for us to delineate whether mimicry develops longitudinally across the lifespan, and, importantly, if there are any important developmental transitions. It would be a fruitful avenue for further research to investigate mimicry using a wider variety of age ranges, especially in the case of orangutans, where the bias towards the testing of adults in mimicry studies is even more extreme.

Another limitation concerns the age difference between the individuals depicted in the stimuli and the participants (both human and orangutan). The stimuli featured human children and juvenile orangutans, while the observers were human adults and infants and mostly adult orangutans. Although this choice was largely driven by practical considerations, since finding play face stimuli with adult orangutans proved challenging, it nonetheless limits the generalizability of our findings. Future research should therefore aim to include age-matched and cross-age stimuli to better disentangle the effects of age and species on play face mimicry. A final limitation of note is that we only tested the mimicry of one expression. We chose to examine the mimicry of play faces given the extensive literature on this expression and its relevance for social behaviours in both humans and non-human animals [10,15]. Additionally, play faces are widespread in many taxa, making these a good candidate to examine the phylogenetic origins of mimicry. Although the attentional capacities of young infants and orangutans prevented us from examining more expressions, it will nonetheless be important for future studies to test the mimicry of a wider range of emotional expressions, using a similar paradigm to our own. In addition, the use of temporally disjointed stimuli, in which the interaction partners in the stimuli show play faces (or other emotions) with a brief delay, may further help to test whether mimicry depends on perceiving the interaction as causally coherent. These additional manipulations will help better delineate the functional boundaries and prevalence of mimicry in non-human animals.

### (d) Conclusion

The results of this study suggest that human infants and adults reliably mimic the play face expressions of conspecifics. Additionally, we found evidence of an important developmental difference—infants expressed robustly more play faces in response to our stimuli compared to adults. This was not the case for smiling behaviours, in which adults’ frequency of smiling far surpassed that of infants. Interestingly, we found no clear evidence of play face mimicry in our orangutan sample, although we wish to stress that this was probably owing to our experimental set-up and the nature of our task, where live social interaction was absent, rather than the fact that this phenomenon does not exist in this species. Our study was unique in its ability to examine mimicry-related responses at the intersection of ontogeny and phylogeny. This approach highlights the value of expanding samples across age groups, in both human and non-human animal work. To this end, our findings highlight some important developmental differences in facial expression mimicry in humans, while similarly emphasizing some potential cross-species differences in the boundaries of mimicry response patterns. Overall, this study contributes to our understanding of how the production of emotions and mimicry changes across human development and between species. However, it also emphasizes a need for further research to disentangle the complexities surrounding mimicry across species.

## Supplementary Material



## Data Availability

We report how we determined our sample size, all data exclusions (if any), all manipulations, and all measures in the study, and the study follows JARS95. All non-identifiable data, analysis code, and research materials are available at [118]. Supplementary material is available online [119].

## Theme

One contribution of 19 to a theme issue ‘Mechanisms, development, phylogeny and functions of emotional expressions’.
