# Supplementary material for: The development of play face mimicry: comparative insights from human infants, adults and orangutans
Source: Philos Trans R Soc Lond B Biol Sci. 2026 Sep 24;381(1959):20250132. doi: 10.1098/rstb.2025.0132 (PMC13601888; doi:10.1098/rstb.2025.0132)
Supplement: Supplementary file 1 [file rstb.2025.0132.zip › rstb.2025.0132/rstb20250132_si_001.docx]

**Supplementary Materials 1 – Methodological Information**

**Participants**

Participants were *n* = 36 human adults, *n* = 77 human infants, and *n* = 6 orangutans (5 adults, 1 juvenile). See Table S1 for an overview of the demographic information and Table S2 for an overview of orangutan participants. We based our human adult sample size on an a-prior power calculation using PANGEA (Westfall, 2015), based on a two-level mixed design, with trials nested within participants (although we ended up opting for Bayesian statistics for our analyses, see a detailed explanation below). This resulted in a minimum sample size of *N* = 30 to detect a medium effect size *d* = 0.45 with 80% power. We collected an additional *n* = 6 participants to account for the possibility that some of the sessions would not be completed successfully. The sample size for human infants and orangutans was not pre-determined and was collected opportunistically based on willingness to participate. Adult participants were mostly students and were recruited in pairs via the Leiden University research participation website (SONA) and rewarded with research credits or €3,50 for their participation. Infants were recruited and tested at the University of Amsterdam, as part of a longitudinal study on the development of the social self and rewarded with a toy and a “Young Scientists” certificate for their participation. Mothers of infants aged 0-6-months were recruited via social media and via flyers hung in public spaces. For this study, we used data from the second measurement occasion, when infants were 15 months old. The adult and infant studies were approved by the ethical review boards of Leiden University (#3152) and the University of Amsterdam (FMG-970), respectively. All testing was conducted in accordance with the Declaration of Helsinki.

Orangutans were housed at two different zoos in the Netherlands: Apenheul (*n* = 4) and Ouwehands Dierenpark (*n* = 2). Although there was a total of 15 orangutans housed at both locations, only six of these individuals were interested in participation, which occurred on a voluntary basis. Group composition differed across sites, and this impacted the procedures of the experiment. In Ouwehands Dierenpark, orangutans lived in three groups, with Anak being separated from all other individuals out of concern for her wellbeing due to her advanced age. The groups lived in and alternated between three indoor enclosures (total area 370m^2^) and one outside enclosure (total area 348m2). At Apenheul, the eight orangutans were not arranged in fixed groups, but rather the housing combinations changed regularly. Orangutans at Apenheul were housed in four indoor enclosures (total area 232m^2^), with each attached to an outdoor island network (total area 1800m^2^). At both sites, orangutans were provided with enrichment, including climbing ropes and wooden constructions in their enclosures.

Orangutans at both sites were habituated to visitors and familiar with researchers. Before we conducted our experiment, a training period was conducted to familiarize the orangutans to the research setup. Participation was strictly voluntary: individuals were never forced, rewarded, or separated from their groups. The study was conducted in adherence with the Ex Situ Program (EEP) guidelines of the European Association of Zoos and Aquaria (EAZA).

***Data Exclusions*.** A total of *n* = 14 infants were excluded from the experiment due to fussiness or technical errors. As indicated in detail below, *n* = 6 adult participants correctly identified the aims of the study and we, as such, decided to run the analyses with these participants included and excluded, which did not change our results. As such, we report the results of our analyses with these participants included.

**Design**

Since we analyzed human and orangutan data separately, the design of the study was slightly different across groups. In humans, the present study had a between-subjects design with one between subjects factor: group membership (2 levels: human adult, human infant) and one within-subjects’ factor: stimulus type (2 levels: control stimulus [in which no play or laughter face was visible] and experimental stimulus [in which a play or laughter face was clearly visible]). For the orangutans, we retained only a single within-subjects factor—stimulus type—since we did not compare responses between juveniles and adults. Owing to the differing attentional capacities of each group and, in the case of orangutans the opportunity to test individuals over multiple days, participants completed a different number of trials. Human adults completed *n* = 32 trials, human infants completed *n* = 8 trials, and orangutans completed an average of *n* = 15.33 trials spread over 34 days.

**Materials**

***Human adults and infants.*** The stimuli used in the present study were four-second video fragments gathered from the Dutch public television network (NPO) and YouTube. Stimuli consisted of two children engaged in an interaction with one another, presented in full color without sound. In experimental trials, two children were shown smiling and at least one child displayed a smile with a jaw drop (i.e., a play face: Action Units 12 + 25 + 26). In control trials, the children showed only neutral facial expressions. Importantly, to limit the extent to which the participant felt like they were involved in the interaction (which may be heightened with stimuli displaying direct eye contact; the individuals in the stimuli made no direct eye contact with the participant. For adults, stimuli were displayed on a 47” television (LG 47LH5000), and, for infants, on a 30 by 30 cm computer screen. For adults, video data were recorded using two Sony HDR-CX240 Handycams. For infants, we used a lab observation system, Viso (Noldus, Wageningen), which had four wall-mounted cameras, one on each wall of the room. We used the camera that directly faced the seated infant for our analyses.

***Orangutans.*** The stimuli used for the orangutan portion of the study were four-second video fragments gathered from YouTube. Several primer videos (e.g., of wild animals, a man cleaning a window) were shown before the experimental trials to attract the orangutan’s attention and motivate them to sit in front of the television. Stimuli consisted of two to three juvenile individuals engaged in interaction with a conspecific, presented in full color without sound. In experimental trials, at least one individual displayed a play face (Action Units 10 + 12 + 25 + 26). In control trials, the individuals showed only neutral facial expressions, although in one control trial one individual also displayed a jaw drop (Action Units 25 + 26, without the presence of Action Units 10 and 12). As in the human stimuli, the individuals did not gaze directly into the camera, so as not to give the individual the feeling of involvement in the interaction. The stimuli were displayed on a full screen on a television, affixed to the outside of the enclosure, facing the orangutans. Orangutans’ reactions to the stimuli were recorded with three cameras, a Sony DCR-SX45 Handycam, a Sony HDR-CX560 Handycam, and a Logitech C920 HD Webcam). One Handycam was placed on a tripod so that it could be used to manually follow an individual, while the other Handycam was placed below the television. The webcam was placed centrally above the television.

**Procedure**

***Human adults and infants.*** The experiment with human adults took place at the labs of Leiden University. After welcoming the participants in groups of two, the participants reviewed the information brochure and signed the consent forms. Because participants may have modified their behavior if they were privy to the aims of the experiment (measuring facial mimicry), in the information brochure they were simply told that the experiment aimed to measure their “attention” while viewing video scenes. After providing consent, the participants were asked to sit on chairs approximately 1-meter distance to the television screen, in front of a visible recording device. After providing some demographic details, the experiment began.

Human adults saw a total of *n* = 32 trials, divided into four blocks of *n* = 8. The same stimuli were displayed in each block, but the order of control and experimental trials was counterbalanced across blocks. Each block contained an equal number of control (*n* = 4) and experimental (*n* = 4) trials. In each trial, one of the eight stimuli was displayed and repeated four times within each trial. Each trial started with a fixation cross and an auditory bell; between each presentation of the stimulus a blue screen was interspliced (Figure S1). One trial took approximately 19 seconds, and the total experiment took approximately 15 minutes to complete. After viewing all the stimuli, the experiment was concluded. Since deception was used, the researcher asked each participant whether they were aware of the aims of the study and noted any participants that indicated this.

For human infants, the procedure was very similar. Infants and their mothers were invited to the labs of the University of Amsterdam. After reading the information brochure and signing the consent forms, the mother and infant pairs were asked to sit in front of a computer screen at approximately 1 meter distance and view one block of trials identical to those used in human adults. The infants sat on a high-chair next to the mother, but in the case that an infant became fussy or agitated they were permitted to sit on their mother’s lap. Mothers were given instructions to watch the videos with their infants and asked simply to act as they normally would with their infant. After viewing the eight trials, the experiment was concluded. In total, this took approximately four minutes.

***Orangutans.*** As indicated, a training period preceded the experiment itself to familiarize the orangutans with the setup and to capture their interest. Stimuli used in the training period were not of primates and were presented on the same screen as in the experiment itself. To start the experiment, the television screen was placed in front of one of the enclosures. If the individuals in said enclosure were not interested, or unwilling to participate, the screen was moved to the next enclosure. The screen was easily visible and accessible for all subjects. To ensure the focal individual’s face was visible (i.e. oriented towards the camera and not obstructed by a food item), the experiment was started only if no play or feeding behaviors occurred within the preceding five minutes.

After an orangutan approached the television screen, one of the primer videos was displayed. If the individual sat in front of the screen for more than three uninterrupted seconds, an experimental trial began. Unlike in the human experiment, a trial consisted of one video display (control or experimental), followed by an observation period of 3.5 minutes. As with the human experiment, a single trial consisted of four iterations of the same short segment (4 seconds), with a blue screen interspliced between each segment (1 second). As such, each trial took approximately 19 seconds to complete (Figure S1). If the individual did not attend to at least one of the four segments, the trial was considered invalid and presented again at a later opportunity. Sessions consisted of one experimental trial and one control trial; with the order of both counterbalanced between sessions. After one block of four sessions, the order of the presentation of the stimuli was changed again. Individuals participated in a maximum of three sessions per day, with a minimum break of 30 minutes between sessions.

**Figure S1.** Trial Structure

Note. Each trial consisted of 4 repetitions of the same 4-second-long video fragment followed by a 1-second-long presentation of a blue screen, for a total trial length of 20 seconds. Trial structure was the same for all 3 experiments and all participants were shown videos of conspecifics (i.e. human adults and human infants saw the same stimuli containing human children, while orangutans saw videos containing juvenile orangutans).

**Statistical Analyses**

***Scoring behaviors.*** Human behaviors were coded manually trial-by-trial by a certified FACS coder (CR). The first authors TZ and CR coded 20% of the total videos together to ensure sufficient inter-rater reliability, which was good (Cohen’s kappa > 0.77) for all behaviors. Orangutan behaviors were coded by author TZ with the help of an experienced research assistant. Behaviors were coded as present or absent in a particular trial. Of central relevance to our research question was the play face, in humans, which we defined as a smile (activation of the lip corner puller, *zygomaticus major*, AU12), combined with a parting of the lips (AU25) and dropping of the jaw (AU26). In line with previous work on orangutans, we defined a play face as activation of AUs 10 + 12 + 25 + 26 (or 27), and a bared teeth display as activation of AUs 10 + 12 + 25 (Pritsch, Telkemeyer, Mühlenbeck, et al., 2017). Importantly, we distinguished between different antecedents of play faces to be sure that our results captured true mimicry processes, rather than emotionally congruent facial displays caused by some other phenomenon. For example, participants’ play faces may have been elicited by interaction with their partner, rather than by the observation of the stimulus material. As such, we scored these “unrelated” instances separately and excluded these trials from our analyses. Additionally, given that smiling in humans may be viewed as a pre-cursor or low intensity version of laughter (Davila-Ross & Dezecache, 2021b; Haakana, 2010) and as we saw many human participants smile in response to our stimuli, we also examined instances of smiling behaviour (i.e., activation of the *zygomaticus major* without the parting of the lips and dropping of the jaw). In order to compare across species, we also coded orangutan “bared teeth” displays (involving AU10 + 12 + 25), since these have been argued to be homologous to human smiles.

In addition to play faces and smiles, the lead authors and a group of research assistants also coded several other behaviors which we deemed potentially relevant based on previous research. These additional behaviors (along with a short description of each) are listed in Table S3. Note that since the interaction context was different between all groups (e.g., infants were seated next to their mother, adults sometimes next to a friend or stranger, orangutans sometimes in groups) we tried to control for the different behaviors in each specific setting that may have influenced participants’ reactions to the stimuli. As such, the coding schemes for all groups differed. For orangutans, we included other expressions that could potentially signal an emotional reaction (scratching, nose wiping, and yawning).

Lastly, since participation by the orangutans was unrewarded and not actively encouraged (unlike the participation of the human participants), there were differences in the amount of time spent looking at the stimuli between trials. Looking time per trial (in seconds; maximum of 20sec) was scored manually based on visual inspection for all recorded orangutan trials, to investigate whether the experimental or control stimuli were attended to more than the other category.

***Modelling.*** We performed all analyses using R Studio Version 3 (R Studio, 2023). To analyze the presence of play faces and smiles (which were coded as 1 = ‘present’, 0 = ‘absent’ in any given trial), as well as to provide evidence for the null hypothesis in the event of any non-significant findings using frequentist frameworks, we ran a series of Bayesian generalized linear mixed models with Bernoulli distributions in the *brms* package (Bürkner, 2018). We ran all models with 4 chains and 5000 iterations (including 1000 warm-up iterations). As per the recommendations of Depaoli and van de Schoot (2017), we examined model convergence by visual inspection of the trace plots and histograms of the posteriors as well as inspection of the Gelman and Rubin diagnostics. Since we had only limited studies to base our prior estimates on (many of which used drastically different methodologies), we used the default *brms* priors for all models; flat priors for all fixed effects and Student’s *t* priors with 3 degrees of freedom for the Intercept and other variance parameters.

In the results, we report model estimates (including 95% credible intervals [CIs]) and the probability of direction (*pd*), which we calculated using the *bayestest R* package (Makowski et al., 2019a). Probability of direction values represent the proportion of the posterior distribution that is the same as the median’s sign. Thus, this value is a measure of certainty of the directionality of the effect (i.e., whether it is positive or negative), and ranges from 0.50 to 1.00 (Makowski et al., 2019b). Additionally, we used the *emmeans* package (Lenth et al., 2019) to run pairwise comparisons on the posterior predictive distributions to better describe the effects, paying particular attention to whether the highest posterior densities (HPDs) crossed zero. To aid interpretation, we took the exponent of the model coefficients (*b*) to interpret the results of the model at the odds ratio level. Odds ratios greater than 1 indicate that the chance of the presence of a certain behavior (e.g., producing a play face) increase across levels of the predictor, whereas odds ratios less than 1 indicate that the chance of the same behavior decrease across levels of the predictor.

**Human infants and adults**. For human infants and adults, we constructed separate models for play faces and smiles, all of which had the same structure. Firstly, we ran two intercept-only models which predicted both play faces and smiles, allowing the intercept to vary per participant. We used this model to determine variance ratio (the proportion of the total variance explained by both fixed and random effects) using the “variance_decomposition” function in the *performance* package (Lüdecke et al., 2021). In all cases, examination of the variance ratio revealed that there was sufficient variance at the level of the participant (level 2), which suggested that mixed models were suitable. Secondly, we ran two models which included the effect of *condition* (experimental trial vs. control) as a predictor of play faces and smiles. Thirdly, since the stimuli used in the infant and adult study were identical, we decided to directly compare infants and adult mimicry behavior in a single analysis. Here, we used the first 8 trials of the adult data and ran a model which included *condition* (experimental vs. control) and *age* (infant vs. adult) as predictors of play faces and smiling. This allowed us to compare any mimicry of play faces and smiles across age groups to examine any between-group differences.

Finally, after comparing adults and infants directly, we ran two models which included the effect of *condition*, as well as the effects of other potentially influential behaviors as covariates (to examine whether the effect of *condition* became weaker or disappeared). For infants, we included the presence of *parental positive vocalizations*, *parent laugh faces* and *parent pointing* behaviors as covariates. For adults, we included *partner familiarity* (known vs. unknown), *sex*, and *partner eye contact* as covariates. Details of these control behaviors are highlighted in the ethogram in Appendix 1. For all models, we allowed the intercept to vary by participant.

**Orangutans.** For orangutans, we approached the analysis differently. To foreshadow our results, there was only one orangutan play face display, and zero bared teeth displays, across all the valid trials. This made statistical analysis of this data impossible, and, rather, we provide a descriptive account of our findings. The time spent looking at the stimuli per trial was compared between the play and control condition using a Wilcoxon rank sum test, since the data did not satisfy the criteria for a parametric test.

**Table S1.** Demographic Information

| **Group** | ***M*_age_** | ***SD*_age_** | **Age Range** | ***%* Female** |
| --- | --- | --- | --- | --- |
| Human adults | 26.09 years | 12.48 | 19-66 years | 69.4% |
| Human infants^1^ | 14.91 months | 0.69 | 12-16.31 months | 40.42% |
| Orangutans^2^ | 39.33 years | 13.70 | 5-45 years | 50% |

^1^*Note.* Only *n* = 47 parents filled in the demographic questionnaire

**Table S2.** Demographic Information Orangutan Participants

| **Subject** | **Sex** | **Age (years)** | **Zoo** | ***n*_trials_** |
| --- | --- | --- | --- | --- |
| Amos | Male | 21 | Apenheul | 5 |
| Anak | Female | 45 | Ouwehands | 10 |
| Kevin | Male | 40 | Apenheul | 18 |
| Sabar | Male | 5 | Ouwehands | 17 |
| Sandy | Female | 39 | Apenheul | 10 |
| Wattana | Female | 26 | Apenheul | 32 |

*Note.* This table provides an overview only of the orangutans that participated in the study. The actual subject pool was much larger, as orangutans were not coerced or forced in any way to participate.

**Table S3.** Trial Breakdown for Human Participants

|  | **Trial 1** | **Trial 2** | **Trial 3** | **Trial 4** | **Trial 5** | **Trial 6** | **Trial 7** | **Trial 8** |
| --- | --- | --- | --- | --- | --- | --- | --- | --- |
| **Repetition 1** | E (1) | C (1) | C (2) | E (2) | E (3) | C (3) | C (4) | E (4) |
| **Repetition 2** | E (2) | C (2) | C (1) | E (1) | E (4) | C (4) | C (3) | E (3) |
| **Repetition 3** | E (4) | C (4) | C (3) | E (3) | E (2) | C (2) | C (1) | E (1) |
| **Repetition 4** | E (3) | C (3) | C (4) | E (4) | E (1) | C (1) | C (2) | E (2) |

*Note*. E = experimental, C = control trial. In each trial, one of four stimulus types were shown.

**Table S4.** Ethogram of Behaviors Coded Across Experimental Groups (Human Adults, Human Infants, Orangutans)

| **Group** | **Behavior** | **Description** |
| --- | --- | --- |
| **Both human groups** |  |  |
|  | Stimulus smile | Activation of zygomaticus major (AU12) occurring after viewing stimulus material |
|  | Play face | Activation of zygomaticus major (AU12) and jaw drop (AU 25 + 26) occurring after viewing stimulus material |
|  | Spontaneous smile | Activation of zygomaticus major (AU12) occurring spontaneously |
|  | Partner smile | Activation of zygomaticus major (AU12) occurring after viewing with partner |
|  | Laughter vocalization | Audible laughter vocalization |
|  |  |  |
|  | Looking at partner | Participant looks in the direction of the partner |
| **Human adults** |  |  |
|  | Non-laughter vocalization | Vocalization other than laughter (e.g., sigh, moan, grunt) |
|  | Scratching | Participant scratches body and/or face |
|  | Yawning | Stretch of the jaw with deep inhale, short exhale and closing of jaw |
|  | Head movement | Any movement of head from the neutral position |
|  | Nose touching | Any touching of the nose region |
| **Human infants** |  |  |
|  | Pointing to stimulus | Mother and/or child points in the direction of the stimulus |
|  | Positive vocalization | Mother produces vocalization in combination with activation of zygomaticus major (AU12) |
| **Orangutans** |  |  |
|  | Play face | Activation of AUs 10 + 12 + 25 + 26 (or 27) |
|  |  |  |
|  | Bared teeth display | Activation of AUs 10 + 12+ 25 |
|  |  |  |
|  | Scratching | Animal scratches itself anywhere on the body |
|  |  |  |
|  | Yawning | Stretch of the jaw with deep inhale, short exhale and closing of the jaw |
|  |  |  |
|  | Play | Animal engages in play behaviors (e.g., rolling over, play fighting with another individual) |
|  |  |  |
|  | Nose wipe | Animal makes contact with nose using its fingers |

**References**

Pritsch, C., Telkemeyer, S., Mühlenbeck, C., & Liebal, K. (2017). Perception of facial expressions reveals selective affect-biased attention in humans and orangutans. Scientific Reports, 7(1), 7782. https://doi.org/10.1038/s41598-017-07563-4

Davila-Ross, M., & Dezecache, G. (2021b). The Complexity and Phylogenetic Continuity of Laughter and Smiles in Hominids. Frontiers in Psychology, 12. https://doi.org/10.3389/fpsyg.2021.648497

Haakana, M. (2010). Laughter and smiling: Notes on co-occurrences. Journal of Pragmatics, 42(6), 1499–1512. https://doi.org/10.1016/j.pragma.2010.01.010

Westfall, Jacob. 2015. “PANGEA: Power Analysis for General Anova Designs.” Unpublished Manuscript. Available at <Http://Jakewestfall.org/Publications/Pangea.pdf>.

Citing RStudio. Posit Support (2023).

Bürkner, P.-C. Advanced Bayesian Multilevel Modeling with the R Package brms. The R Journal 10, 395 (2018).

Depaoli, S. & van de Schoot, R. Improving transparency and replication in Bayesian statistics: The WAMBS-Checklist. Psychol Methods 22, 240–261 (2017).

Makowski, D., Ben-Shachar, M., & Lüdecke, D. (2019a). bayestestR: Describing Effects and their Uncertainty, Existence and Significance within the Bayesian Framework. Journal of Open Source Software, 4(40), 1541. https://doi.org/10.21105/joss.01541

Makowski, D., Ben-Shachar, M. S., & Lüdecke, D. (2019b). bayestestR: Describing Effects and their Uncertainty, Existence and Significance within the Bayesian Framework. Journal of Open Source Software, 4(40), 1541. https://doi.org/10.21105/joss.01541

Lenth, R., Singmann, H., Love, J., Buerkner, P., & Herve, M. (2019). Package ‘emmeans.’

Lüdecke, D., Ben-Shachar, M. S., Patil, I., Waggoner, P., & Makowski, D. (2021). performance: An R Package for Assessment, Comparison and Testing of Statistical Models. Journal of Open Source Software, 6(60), 3139. https://doi.org/10.21105/joss.03139
