# Supplementary material for: The development of play face mimicry: comparative insights from human infants, adults and orangutans
Source: Philos Trans R Soc Lond B Biol Sci. 2026 Sep 24;381(1959):20250132. doi: 10.1098/rstb.2025.0132 (PMC13601888; doi:10.1098/rstb.2025.0132)
Supplement: Supplementary file 1 [file rstb.2025.0132.zip › rstb.2025.0132/rstb20250132_si_002.docx]

**Supplementary Materials 2 – Additional Results**

**Table S5.** Count Data Displaying Number of Trials in Which Human Infants, Human Adults and Orangutans Displayed Play Faces, Collapsed Across Experimental/Control Trials

|  | **Human Infants** | | **Human Adults** | | **Orangutans** | |
| --- | --- | --- | --- | --- | --- | --- |
| **Play Face** | **Present** | **Absent** | **Present** | **Absent** | **Present** | **Absent** |
| **Trial Type** |  |  |  |  |  |  |
| **Control** | 71 | 168 | 21 | 547 | 0 | 45 |
| **Experimental** | 108 | 136 | 34 | 534 | 1 | 46 |

*Note. Number of trials was not consistent across groups. Human infants completed n* = *8 trials, human adults completed n* = 32, *and orangutans completed an average of n* = 15.33.

**Table S6.** Count Data Displaying Number of Trials in Which Human Infants and Human Adults Smiled and Orangutans Showed a Bare Teeth Display, Collapsed Across Experimental/Control Trials

|  | **Human Infants** | | **Human Adults** | | **Orangutans** | |
| --- | --- | --- | --- | --- | --- | --- |
| **Smile/Bared Teeth** | **Present** | **Absent** | **Present** | **Absent** | **Present** | **Absent** |
| **Trial Type** |  |  |  |  |  |  |
| **Control** | 49 | 190 | 106 | 462 | 0 | 45 |
| **Experimental** | 51 | 193 | 179 | 389 | 0 | 46 |

*Note. Number of trials was not consistent across groups. Human infants completed n* = *8 trials, human adults completed n* = 32, *and orangutans completed an average of n* = 15.33.

**Table S7.** Model Predicting Play Face Display in Infants by Condition

|  | **Play Face** | |
| --- | --- | --- |
| *Predictors* | *Odds Ratios* | *CI (95%)* |
| Intercept | 0.26 | 0.14 – 0.46 |
| Condition: Experimental | 2.68 | 1.68 – 4.39 |
| **Random Effects** | | |
| σ^2^ | 3.29 | |
| τ_00_ _BabyID_ | 3.29 | |
| ICC | 0.50 | |
| N _BabyID_ | 63 | |
| Observations | 483 | |
| Marginal R^2^ / Conditional R^2^ | 0.042 / 0.354 | |

**Table S8.** Model Predicting Play Face Display in Adults by Condition

|  | **Play Face** | |
| --- | --- | --- |
| *Predictors* | *Odds Ratios* | *CI (95%)* |
| Intercept | 0.00 | 0.00 – 0.01 |
| Condition: Experimental | 2.46 | 1.18 – 5.33 |
| **Random Effects** | | |
| σ^2^ | 3.29 | |
| τ_00_ _Participant_ | 13.94 | |
| ICC | 0.81 | |
| N _Participant_ | 36 | |
| Observations | 1136 | |
| Marginal R^2^ / Conditional R^2^ | 0.000 / 0.445 | |

**Table S9.** Model Predicting Smiling in Infants by Condition

|  | **Smiling** | |
| --- | --- | --- |
| *Predictors* | *Odds Ratios* | *CI (95%)* |
| Intercept | 0.18 | 0.10 – 0.29 |
| Condition: Experimental | 1.02 | 0.62 – 1.66 |
| **Random Effects** | | |
| σ^2^ | 3.29 | |
| τ_00_ _BabyID_ | 1.58 | |
| ICC | 0.32 | |
| N _BabyID_ | 63 | |
| Observations | 483 | |
| Marginal R^2^ / Conditional R^2^ | 0.001 / 0.177 | |

**Table S10.** Model Predicting Smiling in Adults by Condition

|  | **Smiling** | |
| --- | --- | --- |
| *Predictors* | *Odds Ratios* | *CI (95%)* |
| Intercept | 0.17 | 0.10 – 0.26 |
| Condition: Experimental | 2.32 | 1.71 – 3.17 |
| **Random Effects** | | |
| σ^2^ | 3.29 | |
| τ_00_ _Participant_ | 1.45 | |
| ICC | 0.31 | |
| N _Participant_ | 36 | |
| Observations | 1136 | |
| Marginal R^2^ / Conditional R^2^ | 0.024 / 0.192 | |

**Table S11.** Model Predicting Play Face Presence by Condition and Age Group

|  | **Play Face** | |
| --- | --- | --- |
| *Predictors* | *Odds Ratios* | *Credible Interval (95%)* |
| Intercept | 0.01 | 0.00 – 0.02 |
| Condition |  |  |
| *Control (Reference)* |  |  |
| *Experimental* | 3.98 | 1.11 – 16.11 |
| Age |  |  |
| *Adult (Reference)* |  |  |
| *Infant* | 46.73 | 10.07 – 275.89 |
| Condition x Age | 0.69 | 0.16 – 2.72 |
| **Random Effects** | | |
| σ^2^ | 0.03 | |
| τ_00_ | 0.16 | |
| ICC | 0.18 | |
| N _Participant_ | 99 | |
| Observations | 771 | |
| Marginal R^2^ / Conditional R^2^ | 0.135 / 0.440 | |

**Table S12.** Model Predicting Smile Presence by Age and Condition

|  | **Smiling** | |
| --- | --- | --- |
| *Predictors* | *Odds Ratios* | *Credible Interval (95%)* |
| Intercept | 0.24 | 0.14 – 0.41 |
| Condition |  |  |
| *Control (Reference)* |  |  |
| *Experimental* | 2.41 | 1.39 – 4.26 |
| Age |  |  |
| *Adult (Reference)* |  |  |
| *Infant* | 0.80 | 0.40 – 1.60 |
| Condition x Age | 0.42 | 0.20 – 0.87 |
| **Random Effects** | | |
| σ^2^ | 0.02 | |
| τ_00_ | 0.17 | |
| ICC | 0.10 | |
| N _Participant_ | 99 | |
| Observations | 771 | |
| Marginal R^2^ / Conditional R^2^ | 0.034 / 0.160 | |

**CONTROL MODELS**

As indicated, there were several behaviors that could have influenced human participants’ propensity to mimic the stimuli displayed. To control for this, we ran models for infants and adults separately which predicted play faces and smiles, while including these potentially influential behaviors as covariates. Examination of the infant model which controlled for the effects of parent positive vocalizations, parent laugh face and parent pointing behaviors revealed that the effect of condition on play face displays was robust and remained influential, but was slightly weaker (*b* = 0.66, 95% CI = [0.15, 1.18], OR = 1.94, *pd* = 0.99) (Table S13). Additionally, in this model, mother play face was also found to be a meaningful predictor of infants’ play faces (*b* = 1.28, 95% CI = [0.68, 1.92], OR = 3.59, *pd* = 1.00). This indicates that the odds of infants’ producing a play face increased by a factor of 3.60 when mothers themselves displayed a play face. Importantly, this was not due to infants’ mimicry of the parents’ facial behavior, as we only considered play faces that occurred as a response to the stimulus in this analysis (and not as a response to interaction with the mother or spontaneously occurring). No other covariates meaningfully influenced infants’ play face behaviors (all 95% CIs spanned zero).

Examination of the adult model which controlled for partner eye contact, gender, and partner familiarity revealed that, when controlling for these additional explanatory variables, the effect of condition remained a meaningful predictor of play faces, *b* = 0.91, 95% CI = [0.17, 1.69], OR = 2.48, *pd* = 0.99 (Table S14). No other variables robustly explained adults’ play face behaviors (all 95% CIs spanned zero). As condition was not a robust predictor of smiling behavior in infants, we performed the control model for adults only. In this model, condition remained a meaningful predictor of smiling behavior, *b* = 0.89, 95% CI = [0.57, 1.22], OR = 2.44, *pd* = 1.00 (Table S15). As with the play face control model, partner eye contact, partner familiarity and gender were not meaningfully associated with smiling. All in all, the results of these analyses suggest that our above findings hold when considering these potentially influential variables, suggesting that these are robust.

**Table S13.** Model Predicting Infant Play Faces from Condition, Parent Positive Vocalizations, Parent Play Face, Parent Pointing Behaviors

|  | **Play Face** | |
| --- | --- | --- |
| *Predictors* | *Odds Ratios* | *CI (95%)* |
| Intercept | 0.10 | 0.05 – 0.22 |
| Condition: Experimental | 1.94 | 1.16 – 3.27 |
| Parent Positive Vocalizations | 1.26 | 0.64 – 2.48 |
| Parent Play Face | 3.59 | 1.96 – 6.80 |
| Parent Pointing Behaviors | 1.45 | 0.83 – 2.56 |
| **Random Effects** | | |
| σ^2^ | 3.29 | |
| τ_00_ _BabyID_ | 3.05 | |
| ICC | 0.48 | |
| N _BabyID_ | 63 | |
| Observations | 479 | |
| Marginal R^2^ / Conditional R^2^ | 0.120 / 0.381 | |

**Table S14.** Model Predicting Adult Play Faces from Condition, Partner Eye Contact, Gender, and Partner Familiarity

|  | **laugh_face** | |
| --- | --- | --- |
| *Predictors* | *Odds Ratios* | *CI (95%)* |
| Intercept | 0.00 | 0.00 – 0.03 |
| Condition: Experimental | 2.48 | 1.18 – 5.40 |
| Looking at Partner | 0.92 | 0.06 – 29.47 |
| Partner Familiarity | 0.06 | 0.00 – 2.22 |
| Gender Partner | 0.09 | 0.00 – 11.19 |
| **Random Effects** | | |
| σ^2^ | 3.29 | |
| τ_00_ _Participant_ | 13.71 | |
| ICC | 0.81 | |
| N _Participant_ | 34 | |
| Observations | 1080 | |
| Marginal R^2^ / Conditional R^2^ | 0.001 / 0.447 | |

**Table S15.** Model Predicting Adult Smiling from Condition, Partner Eye Contact, Gender, and Partner Familiarity

|  | **Smiling** | |
| --- | --- | --- |
| *Predictors* | *Odds Ratios* | *CI (95%)* |
| Intercept | 0.19 | 0.09 – 0.36 |
| Condition: Experimental | 2.43 | 1.76 – 3.39 |
| Looking at Partner | 0.74 | 0.02 – 7.08 |
| Partner Familiarity | 0.58 | 0.22 – 1.50 |
| Gender Partner | 1.17 | 0.41 – 3.55 |
| **Random Effects** | | |
| σ^2^ | 3.29 | |
| τ_00_ _Participant_ | 1.49 | |
| ICC | 0.31 | |
| N _Participant_ | 34 | |
| Observations | 1080 | |
| Marginal R^2^ / Conditional R^2^ | 0.044 / 0.191 | |
